# Necrotizing fasciitis and septic shock due to streptococcal toxic shock syndrome in an elderly patient: A case report

**DOI:** 10.1002/ccr3.6846

**Published:** 2023-01-19

**Authors:** Akiko Kurachi, Yusuke Ishida, Koichi Nakazawa, Toshio Okada, Takumi Kishida, Hiroyuki Uchino

**Affiliations:** ^1^ Department of Anesthesiology Tokyo Medical University Tokyo Japan

**Keywords:** necrotizing fasciitis, polymyxin B immobilized fiber column direct hemoperfusion, septic shock, streptococcal toxic shock syndrome, *Streptococcus dysgalactiae* subsp. *equisimilis*

## Abstract

Streptococcal toxic shock syndrome (STSS) has a high mortality rate, and most patients die within a few days of onset. We report an elderly patient with STSS, necrotizing fasciitis and septic shock caused by group G streptococcus who was successfully treated with multidisciplinary therapy.

## Abstract 2

The elderly patient suffered from streptococcal toxic shock syndrome and went into shock rapidly and progressively. However, prompt surgical treatment, antimicrobial agents, and blood purification therapy such as polymyxin B immobilized fiber column direct hemoperfusion and continuous hemodiafiltration could have saved the patient's life.

## 1 BACKGROUND

Streptococcal toxic shock syndrome (STSS) is a life‐threatening infection, which usually leads to rapid deterioration of the patient's clinical condition. The mortality rate is approximately 30%, with elderly populations showing remarkably worse prognosis.^1^, ^2^ Pyrogenic toxins produced by streptococcus induce hypercytokinemia, leading to the development of a shock state. Here, we report a case of STSS due to group G streptococcus (GGS). The patient exhibited necrotizing fasciitis and septic shock with unstable hemodynamics and was successfully treated with multidisciplinary therapy, including polymyxin B immobilized fiber column direct hemoperfusion (PMX‐DHP) and continuous hemodiafiltration (CHDF) using a polyacrylonitrile AN69ST membrane. We obtained the patient's written informed consent for the publication of this case report.

## 2 CASE PRESENTATION

A 71‐year‐old woman, 154 cm tall, weighing 46 kg, with a history of hypertension and chronic kidney disease, developed lower leg edema 1 month before hospitalization. She complained of right lower leg pain and a red flare on the day of hospitalization. She visited our emergency room due to the development of dysarthria and gait disturbances. When she arrived at the emergency room, her Glasgow Coma Scale (GCS) score was E4V5M6. However, although she was able to communicate, she had slurred speech and dysarthria. Her pupillary diameter was 3 mm bilaterally, indicating no anisocoria, and the light reflex was normal. Physical examination revealed the following: temperature: 36.9°C, blood pressure: 114/72 mmHg, heart rate: 116 beats/min, respiratory rate: 25 breaths/min, and SpO_2_: 97% (on room air). Electrocardiogram revealed atrial fibrillation. Multiple areas of erythema were found on both the right and left lumbar and lower abdominal areas, right proximal femoral areas, and left proximal inner femoral area. There were blisters, ruptured blisters, swelling, and aching pain on the right inner femoral area.

Laboratory examination at admission revealed a low white blood cell (WBC) count of 1000 cells/mm^3^, elevated C‐reactive protein of 16.7 mg/dl, and elevated procalcitonin level of 30.65 mg/dl, indicating leukopenia with evidence of inflammation and bacterial infection. Her blood urea nitrogen level was 68.9 mg/dl, and creatinine was 7.29 mg/dl, indicating impaired renal function, and prothrombin time (PT) was 19.3 s, PT‐INR was 1.63, activated partial thromboplastin time was 39.2 s, fibrin degradation products were 33.9 μg/ml, and D‐dimer level was 10.08 μg/ml, indicating prolonged coagulation and delayed activation of the fibrinolytic system. Blood gas analysis showed increased lactate levels of 4.59 mmol/L.

**FIGURE 1:**
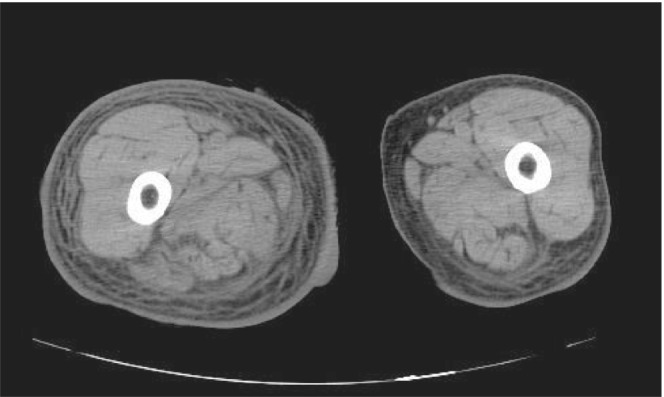
CT scan of the thighs. CT scan of the thighs showed evidence of increased fatty tissue density all around the right thigh.

Brain computed tomography (CT) examination performed to evaluate her neurological status showed an old brain infarction, with no new brain hemorrhage or space occupying lesions. Thoraco‐abdominal and pelvic CT examination displayed predominant right lower leg swelling, subcutaneous edema, and increased adipose tissue signals in the entire right femoral region (Figure 1).

**FIGURE 2:**
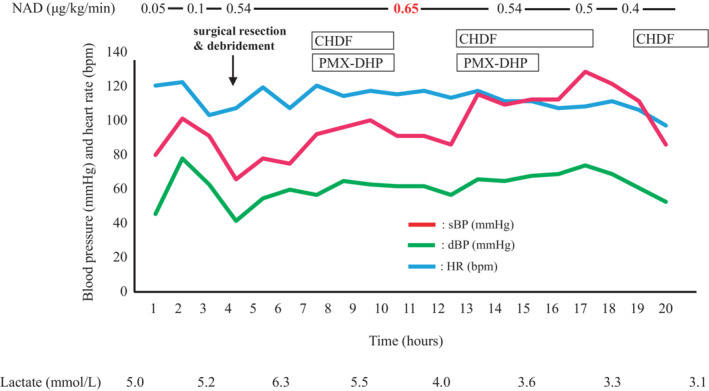
Clinical course after ICU admission. CHDF, continuous hemodiafiltration; dBP, diastolic blood pressure; HR, heart rate; NAD, noradrenaline; PMX‐DHP, polymyxin B immobilized fiber column direct hemoperfusion; sBP, systolic blood pressure

Her blood pressure gradually decreased after hospitalization, requiring vasopressors, and hence, continuous administration of noradrenaline at 0.05 μg/kg/min was initiated. The patient then developed disturbance of consciousness (GCS score was E4V3M4). Based on the clinical, laboratory, and imaging findings, we diagnosed necrotizing fasciitis from the right lumbar to lower abdominal regions along with septic shock. She was admitted to the intensive care unit (ICU) because his blood pressure could not be maintained, and she was also observed to have disturbance of consciousness. At her admission to the ICU, the patient's GCS score was E4V2M2, heart rate was 100 beats/min, blood pressure was 80/63 mmHg, and Sequential Organ Failure Assessment score was 11 points. Her systolic blood pressure subsequently decreased to approximately 60 mmHg, GCS score was E3V1M1, and respiration became unstable. Therefore, we performed endotracheal intubation and commenced mechanical ventilation. Fluid resuscitation was used for shock by administering 1000 ml of extracellular fluid and 500 ml of albumin in ICU. Since her hemodynamic parameters did not respond to fluid loading and noradrenaline administration, the noradrenaline dose was increased to 0.65 μg/kg/min. Figure 2 shows the time course of changes in hemodynamics on ICU admission Day 1. Two hours after ICU admission, we performed right femoral and lower abdominal fasciotomy and purulent drainage. During the procedure, we found right femoral and lower abdominal fascial necrosis. Since the patient also showed deterioration of renal function, we initiated renal replacement therapy. Since endotoxin activity assay at ICU admission showed increased activity levels to 0.7, we performed CHDF using an AN69ST membrane, and PMX‐DHP was added. We subsequently started treatment with two antibiotics: tazobactam/piperacillin and clindamycin (CLDM), and when *Streptococcus dysgalactiae* subsp. *equisimilis* (SDSE) was detected from culture of blood and genital wound samples, we de‐escalated the antibiotics to penicillin‐G on ICU Day 3. Since the patient had wound infection with *Pseudomonas aeruginosa*, ciprofloxacin was additionally administered. Thrombomodulin‐α was administered for the treatment of disseminated intravascular coagulation (DIC) from ICU Day 1 to Day 15. Furthermore, since her urine output was limited to 100 ml/day due to renal failure, continuous high‐flow CHDF was performed with a dialysate fluid rate of 2000 ml/h for 5 days, which was gradually tapered. Due to the substantial drainage from the wound, crystalloid fluid, fresh frozen plasma, and albumin preparations were administered.

**FIGURE 3:**
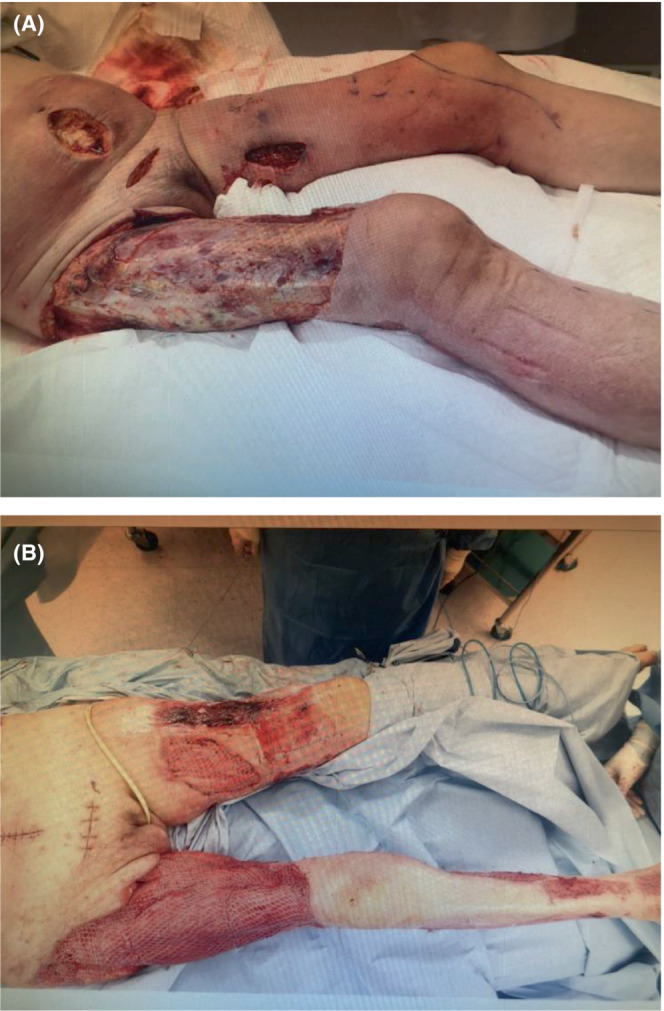
Wound cleaning and debridement. (A) Fasciotomy was performed at the site of erythema in the lower abdomen and proximal right thigh. (B) The wound was cleaned, and debridement was performed daily. This photograph was taken after the procedure on the 10th day after admission to the ICU.

Wound lavage was performed daily, along with blunt scraping of subcutaneous and inter‐fascial tissue and debridement (Figure 3). Thereafter, the patient's hemodynamic condition gradually stabilized, and noradrenaline was tapered and stopped on ICU Day 6. The patient was weaned from mechanical ventilation on ICU Day 27. Her subsequent clinical course was favorable, and she was transferred from the ICU to the regular ward on ICU Day 43.

## 3 DISCUSSION

Initial symptoms of STSS are fever, pain, and swelling of extremities, with rapid clinical deterioration leading to multiple organ failure, including acute respiratory distress syndrome, DIC, and renal disturbance, with extremely high mortality.^2^ While group A Streptococcus (GAS) is a major cause of STSS, cases of group B Streptococcus and GGS‐induced STSS have recently increased.^3^, ^4^ Among GGS, SDSE infection is most frequent in humans.^5^ While STSS due to GAS can occur in patients without underlying conditions,^6^ STSS due to GGS tends to occur in elderly patients and those with underlying conditions, such as diabetes mellitus, cardiovascular diseases, malignancies, cirrhosis, immunosuppressive conditions, or skin diseases.^5^, ^7^, ^8^ In our case, the causative bacterium of STSS was not GAS, but SDSE of GGS. While GGS is a normal inhabitant of the nasopharyngeal cavity, skin and perineal area,^5^ it can cause erysipelas, cellulitis, necrotizing fasciitis, STSS, pneumonia, arthritis, osteomyelitis, meningitis, encephalitis, endocarditis, and sepsis, with clinical presentations similar to GAS. Both group C streptococcus and GGS were previously not considered pathogenic bacteria. However, invasive infection caused by SDSE has been recently recognized, and thus, merits special attention.^9^, ^10^

When STSS is suspected, prompt diagnosis and early initiation of treatment with whole‐body management, antibiotic administration, and surgical intervention are critical.^7^ The primary antibiotic for STSS treatment is penicillin, to which GGS is highly sensitive. Combination therapy with CLDM, which has anti‐exotoxin effects with high tissue penetration properties, is also effective.^5^, ^11^ Reportedly, CLDM also suppresses penicillin‐binding protein synthesis, resulting in enhancement of the penicillin effect in addition to suppression of exotoxin production.^12^ Early debridement is important in cases with necrosis.^7^ Although we did not administer immunoglobulin therapy in our case, it might also be effective for STSS treatment.^5^, ^13^

We used CHDF and PMX‐DHP in addition to hemodynamic management and infection control as a compassionate treatment. CHDF functions as an artificial kidney, removing various mediators of shock. In our case, we used an AN69ST membrane to absorb cytokines. The AN69ST membranes absorb high mobility group box protein 1 (HMGB‐1), which is a late mediator of septic shock.^14^ Previous reports have shown that absorption of HMGB‐1 has beneficial clinical effects in patients with septic shock.^15^, ^16^

PMX‐DHP removes intrinsic cannabinoids, which are critical mediators in the early stages of septic shock and is effective in the treatment of severe sepsis.^17^ In our case, fluid therapy and high doses of noradrenaline were ineffective for treating the shock state, and PMX‐DHP and CHDF enabled the tapering of noradrenaline administration, suggesting that hypercytokinemia might contribute to hemodynamic disruption. We speculate that absorption and removal of cytokines by blood purification therapy using both PMX‐DHP and CHDF resulted in hemodynamic stabilization and the positive outcome in our patient.

Since necrotic tissue has a poor blood supply, penetration of necrotic tissue by antibiotics is generally low. Therefore, early surgical resection of the necrotic tissue to prevent progression of tissue necrosis is prioritized in STSS with comorbid necrotic fasciitis.^7^, ^18^, ^19^ Misiakos et al. reported that the mean interval between diagnosis of necrotizing fasciitis and debridement should be not more than approximately 12 h, since a prolonged duration >12 h worsens mortality.^20^ Bilton et al.^21^ reported that the mortality of patients with necrotic fasciitis who received early and adequate debridement was 4.2%, whereas delayed debridement increased the mortality to 38%. In addition, necrotizing fasciitis causes persistent damage to surrounding tissues via exotoxin secreted by bacteria after establishment of the necrosis, which requires recurrent debridement.^18^, ^19^, ^20^ Our patient underwent emergency debridement within several hours after admission. Furthermore, in our case, daily debridement of necrotic tissue reduced symptoms of infection and successfully preserved the affected leg. With progression of infection, the leg might require amputation. In particular, necrotizing fasciitis cases with comorbid diabetes mellitus have a higher risk of progression of symptoms, requiring amputation of the leg.^22^, ^23^

Here, we reported a case of STSS with necrotizing fasciitis and septic shock. Early multidisciplinary treatment resulted in a favorable outcome despite her advanced age. Many patients with STSS rapidly progress the symptoms which make difficult to rescue. The earliest diagnosis and treatment of STSS are critical. When we see a case with soft tissue infection which has atypical symptoms with rapid progression, STSS should be considered at high priority with proactive treatment.

## AUTHOR CONTRIBUTIONS

**Akiko Kurachi:** Conceptualization; writing – original draft. **Yusuke Ishida:** Conceptualization; writing – original draft; writing – review and editing. **Koichi Nakazawa:** Writing – review and editing. **Toshio Okada:** Writing – review and editing. **Takumi Kishida:** Conceptualization; writing – original draft. **Hiroyuki Uchino:** Writing – review and editing.

## CONFLICT OF INTEREST

The authors declare that they have no competing interests associated with this manuscript.

## CONSENT

Written informed consent was obtained from the patient to publish this report in accordance with the journal's patient consent policy.

## Data Availability

The datasets generated and analyzed during the current study are available from the corresponding author on reasonable request.
